# Effect of In Ovo Administration of a Multi-Strain Probiotic and Zinc Glycine Chelate on Antioxidant Capacity and Selected Immune Parameters in Newly Hatched Chicks

**DOI:** 10.3390/antiox12111905

**Published:** 2023-10-24

**Authors:** Artur Ciszewski, Łukasz S. Jarosz, Arletta Bielecka, Agnieszka Marek, Bartłomiej Szymczak, Zbigniew Grądzki, Anna Rysiak

**Affiliations:** 1 Department of Epizootiology and Clinic of Infectious Diseases, Faculty of Veterinary Medicine, University of Life Sciences in Lublin, Głęboka 30, 20-612 Lublin, Poland; artur.ciszewski@up.lublin.pl (A.C.);; 2 Department of Biochemistry, Faculty of Veterinary Medicine, University of Life Sciences in Lublin, Głęboka 30, 20-612 Lublin, Poland; arletta.bielecka@up.lublin.pl; 3 Department of Preventive Veterinary and Avian Diseases, Faculty of Veterinary Medicine, University of Life Sciences in Lublin, 20-950 Lublin, Poland; agnieszka.marek@up.lublin.pl; 4 Sub-Department of Pathophysiology, Department of Preclinical of Veterinary Sciences, Faculty of Veterinary Medicine, University of Life Sciences in Lublin, Głęboka 30, 20-612 Lublin, Poland; 5 Department of Botany, Mycology, and Ecology, Maria Curie-Skłodowska University, Akademicka 19, 20-033 Lublin, Poland

**Keywords:** chicken, in ovo, multi-strain probiotic, zinc glycine chelate, antioxidant capacity

## Abstract

The aim of this study was to determine the effect of in ovo co-supplementation of chicken embryos with a multi-strain probiotic containing effective microorganisms and zinc glycine chelate on total antioxidant capacity; concentrations of sulfhydryl groups, bityrosine bridges, formylkynurenines, hydroperoxides, proteins, corticosterone, pro- and anti-inflammatory cytokines and heat shock proteins; and the activity of catalase and superoxide dismutase in the serum, yolk sac and tissues of broiler chickens at 12 h and at 7 days after hatching. The results indicate high SOD activity in the small and large intestines of chicks at 12 h post-hatch in the groups receiving the multi-strain probiotic and in the small intestine and yolk sac of birds receiving the multi-strain probiotic and Zn-Gly chelate. High concentrations of TNF-α and IFN-γ in the yolk sac and serum after in ovo administration of Zn-Gly chelate were observed 12 h after hatching. The use of a probiotic and a probiotic with Zn-Gly chelate increased the total antioxidant capacity in the tissues of chickens. It can be concluded that in ovo administration of a multi-strain probiotic and Zn-Gly chelate can maintain the oxidant/antioxidant balance in chickens and increase the defense capacity against oxidative stress.

## 1. Introduction

In intensive poultry production aimed at obtaining high-quality poultry products, it is essential to use healthy chicks with good production traits [1,2]. Embryonic development and the post-hatching period are critical to the survival of chicks, which determines the further course of rearing, the final fattening results and the economic success of the farm.

The intensive development of the chicken embryo and the biological processes and metabolic transformations taking place during this period lead to the formation of reactive oxygen species (ROS) and free radicals, which play an important role in basic biological processes in conditions of heath or disease [3,4]. A disturbance of the balance between the rate of ROS production and the capacity of the antioxidant system in the body results in oxidative stress, which leads to increased embryo mortality, impaired growth and development and a negative impact on the immune processes of birds.

These effects can be counteracted by numerous systems that protect cell components against oxidative damage in chicken embryos and developing chicks [5]. These include antioxidant enzymes, such as superoxide dismutase (SOD), glutathione peroxidase (GSH-PX) and catalase (CAT) [6,7]; water-soluble antioxidants such as vitamin C and glutathione; fat-soluble antioxidants such as vitamin E and alpha-lipoic acid; carotenoids [8,9,10,11], coenzyme Q (CoQ) [12]; and selenium [9]. Essential nutrients for embryonic development are transferred from the feed to the egg yolk and then to the embryonic tissues [5,6,13]. This process is varied, depending in part on the age of the flock of breeding hens, the type of feeding and the composition of the feed, including the use of mineral and vitamin supplements [5,14]. To strengthen antioxidant processes during embryonic development and after hatching, reduce the stress associated with the hatching process, maintain the biochemical and physiological balances in the developing embryo and limit inflammation and the production of cytokines associated with ROS [15,16,17], in ovo methods are used to supply the embryo with compounds acting as antioxidants [18,19,20].

Among such compounds, great interest is currently focused on various probiotics and microelements. The mechanisms of action of probiotics on the body are multi-faceted and not fully explained. The interaction between a probiotic administered in ovo and the intestinal microbiome exerts a modulatory effect on the immune system, stimulating innate and acquired mechanisms of resistance to infection by pathogens, thereby reducing inflammation and oxidative stress [21]. It is worth noting that the use of different probiotic strains in the diet has different effects on the parameters of oxidative stress [22]. For example, in ovo administration of *L. salivarius* and *Pediococcus* spp. increases antioxidant potential, while the use of *Citrobacter freundii* 97A11 intensifies pro-inflammatory reactions and the response to oxidative stress [23,24,25]. In ovo administration of lactic acid bacteria (LAB) has been shown to increase the expression of peroxiredoxin-1 (PRDX1) and superoxide dismutase-1 (SOD1) in intestinal tissue, protecting cells against damage by free radicals and hydrogen peroxide and alleviating symptoms of oxidative stress [23,25].

The term “EM” (effective microorganisms) is often used in the names of products containing microbial strains that do not undergo any technological processing, and published research results indicate a broad range of potential applications of EM in the diet of poultry during growth and rearing [26,27]. However, the literature lacks published results pertaining to the effect of EM on birds following in ovo supplementation.

An important component of probiotic preparations, apart from microorganisms, is chemical elements, especially microelements. Particular attention is focused on the potential use of organic forms of zinc (Zn) in the diet of birds, especially in complexes with glycine, which are more stable and chemically and physically homogeneous than inorganic forms and thus improve the absorption and bioavailability of Zn [28,29,30]. Zinc in the form of glycine chelates has a particularly positive effect on the growth rate of poultry and certain immune parameters [31,32]. These compounds have been shown to stimulate cellular and humoral immune processes; increase the concentrations of cytokines and class A, G and M immunoglobulins in the peripheral blood; and increase the synthesis of acute-phase proteins with immunoregulatory properties in the serum and tissues of poultry [31,32,33].

The literature lacks information on the effect of the combined use of multi-strain probiotics containing effective microorganisms and zinc glycine chelate on antioxidant and immune processes in poultry. In our study, we attempted to verify the hypothesis that co-supplementation with multi-strain probiotics containing effective microorganisms and zinc glycine chelate at 17 days (E17) of embryonic development improves health parameters in hatchlings by enhancing antioxidant capacity and immune functions. The results will provide a better understanding of the role of multi-strain probiotics containing effective microorganisms and zinc glycine chelate in antioxidant and immune processes in chicks in the first few days post-hatch.

## 2. Materials and Methods

### 2.1. Ethical Statement

The experiment was carried out at the Experimental Station of the Poznań University of Life Sciences, Gorzyń 4, Międzychód Commune. All research procedures were approved by the Local Ethics Committee for Animal Testing at the University of Life Sciences in Lublin, Poland (approval No. 106/2022 of 17 October 2022).

### 2.2. Incubation Period

Hatching eggs were obtained from 36-week-old commercial broilers (Ross × Ross 708) and delivered to the Experimental Station of the Poznań University of Life Sciences, ul. Gorzyń 4, Międzychód Commune. The eggs were stored at 21 °C for 24 h. All eggs were individually weighed and marked, and the surface of the eggshells was disinfected with Viron FF (glutaraldehyde, didecyldimethylammonium chloride, quaternary ammonium compounds and benzyl-C12-C16-alkyldimethyl; DDD-1, Bielsko Biała) in 1 mL of L-1 solution. The eggs were incubated in JARSON Model JD-18 incubators (Gostyń, Poland) from day 1 to day 18 at T 37.8 °C, relative humidity (RH) 55–60%. On the 19th day of incubation (DOI), the eggs were transferred to a JARSON Model ATLAS-180 hatching chamber. For the last three days of incubation (19–21 DOI), RH was maintained at 60–65%. On days 7 and 17 of incubation, all eggs were candled and cracked, and unfertilized and dead embryonic eggs were removed.

On day 17 of incubation, 1400 fertile eggs of similar weight were randomly divided into four groups, with 10 replicates per group and 35 eggs per replicate (350 eggs per group) (Table 1). The multi-strain probiotic EM Provet, manufactured by Greenland Technologia EM (Janowiec, Poland), was delivered in powder form. The exact composition of the probiotic is described in the work of Ciszewski et al. [34]. EM Provet was diluted in phosphate-buffered saline (PBS) to obtain 100 µL of a solution containing 1 × 10^5^ CFU *S. cerevisiae*, 1 × 10^7^ CFU *L. casei* and 1 × 10^7^ CFU *L. plantarum*. The cell viability of the probiotic bacteria and their content per gram of product (CFU/g) were tested as described by Weese and Martin [35] in the national reference laboratory of the National Veterinary Research Institute in Puławy, Poland. In addition, the manufacturer of EM Provet had assessed the viability of the probiotic bacteria cells and their content per gram of the product, ensuring that it meets all quality criteria for products containing live bacterial cultures. The Zn glycine chelate was manufactured by ARKOP Sp. z o.o. (Bukowno, Poland). The Zn-Gly powder, containing 250 mg Zn-Gly per g of product, was dissolved in deionized water to obtain solutions containing 100 µg Zn-Gly/100 µL MQ water. All procedures are described in detail by Ciszewski et al. [34].

### 2.3. In Ovo Inoculations

At 17 DOI, all eggs were decontaminated with 75% ethanol and inoculated through the air chamber (using a 2.5 cm long 23G needle) with 500 μL of 0.9% saline or bioactive compound, into the amniotic sac. The puncture sites in the eggs were immediately sealed with sterile paraffin, and the eggs were returned to the incubator. The in ovo injection procedure was performed within 30 min. All methods are described in detail by Alizadeh et al. [36,37] (Table 1).

### 2.4. Birds and Housing

After hatching, 150 day-old chicks from each group were randomly selected for analysis. Each test group had 10 replicates of 15 birds per replicate. The rearing period was 7 days. The birds received a basal diet without bioactive compounds, coccidiostats or antibiotics. They received starter feed S (1–21 days) in the form of crumble. Feed and water were provided as standard. The basal diet was formulated in accordance with nutrition recommendations for Ross 308 broiler chickens (Aviagen, Broiler Ross Nutrition Supplement). The content of nutrients in the diets was calculated from the chemical composition of the feed materials and the metabolic energy value. The basal diet was composed of wheat, soybean meal and maize and contained 22.7% crude protein and 12.47% apparent metabolizable energy corrected to zero nitrogen retention (AMEn). Detailed information is given in the work of Ciszewski et al. [34]. The birds were housed under the same controlled environmental conditions recommended for this line of chickens. The chickens were reared in pens on wood shavings. The pens were equipped with feeding lines and nipple drinkers. Until 7 days of age, the light intensity was 30–40 lux. At that time, the temperature was 31–33 °C and the relative humidity was 60 +/− 10%.

### 2.5. Collection of Blood and Tissue Samples

Peripheral blood samples were collected 12 h after hatching from chicks killed by decapitation (without having received feed or water) and from the wing vein at 7 days after hatching. Tissues were collected at the same times: from the liver, small and large intestines, heart, pectoral muscle and yolk sac at 12 h after hatching and from the liver, small and large intestines, heart and pectoral muscle of the same chickens at 7 days of age. The blood samples were collected in sterile vacuum tubes with a clotting activator (Vacuette, Medlab Products, Raszyn, Poland). Blood and tissues were sampled from three birds from each replicate (30 samples in total) in each experimental group. Blood and tissue samples were not pooled. The blood samples were transported to the laboratory at +4–8 °C within 1 h and centrifuged at room temperature (20–22 °C) for 15 min at 1000× *g*. Serum and tissue samples were stored at −80 °C for analysis. Individual tissue samples (3 g) were homogenized in an Ultra Turrax homogenizer (Ikawerk, Janke, Kunkel, Staufen, Germany) in phosphate buffer (0.1 mol/L, pH = 7.0), Triton X-100 and a protease inhibitor cocktail (Sigma, Poland). The homogenates were centrifuged at 4 °C for 20 min at 6500× *g*. The supernatants were divided into aliquots and stored at −20 °C for subsequent analyses.

### 2.6. Assay of IL-10, IFN-γ, TNF-α, Corticosterone, Hsp 70, Catalase and Superoxide Dismutase (SOD) in Chicken Serum and Tissues

ELISA kits were used to determine IFN-γ, IL-10 and TNF-α (Qayee-Bio, Shanghai, China, No. QY-E80058, QY-E80011 and QY-E80030); corticosterone and Hsp 70 (Biorbyt Ltd., St. Louis, MO, USA No. orb561496 and orb561492); and catalase and superoxide dismutase (SOD) (Cayman Chemical, Ann Arbor, MI, USA No. 707002 and 706002) in the chicken serum and tissues. All assays were performed according to the manufacturer’s instructions. All samples were tested in triplicate.

### 2.7. Total Antioxidant Capacity (T-AOC)

The analysis of total antioxidant capacity (T-AOC) was based on the ferric reducing capacity of the sample as described by Benzie and Strain [38] and Katalinic et al. [39], with some modifications. The mixture, prepared shortly before use, contained 300 mmol/L acetate buffer (pH 3.6), 10 mmol/L 2,4,6 tri-pyridyl-striazine (TPTZ, Sigma, Poznań, Poland) reagent in 40 mmol/L HCl, and 20 mmol/L FeCl_3_ × 6H_2_O solution in distilled water, in proportions of 10:1:1. Each sample in the amount of 25 μL was added to the mixture (2250 μL), and the absorbance was checked at 593 nm. The working mixture alone was used as a control. The absorbance was read again after 10 min of incubation at room temperature. The difference in absorbance at 0 and after 10 min was compared with a standard curve prepared for 10 different dilutions of Fe (II), between 0 and 1000 μmol/L. The absorbance changes were directly related to the “total” reducing ability of the electron-donating antioxidants in the samples. The results were recalculated per protein content in the sample and expressed as μmol/g protein in homogenate tissue. Each determination was performed in duplicate.

### 2.8. Protein Content

Protein content in the experimental and control samples was determined using the Biuret method, using commercially available assay kits (Cormay, Łomianki, Poland) as described by Gornal et al. [40].

### 2.9. Content of Sulfhydryl Groups

Concentrations of sulfhydryl (SH) residues in the experimental and control samples were measured using a spectrophotometric method, as detailed by Rice-Evans et al. [41]. Each sample in the amount of 300 μL was added to 300 μL of 10% (*w*/*v*) sodium dodecyl sulfate (SDS, Sigma, Poznań, Poland) in 10 mmol/L sodium phosphate buffer (pH 8.0) and thoroughly mixed. Next, 2.4 mL of 10 mmol/L sodium phosphate buffer (pH 8.0) was added. A 300 μL volume of a solution prepared by diluting 20 mg of 5,5-dithiobis-2-nitro benzoate (Sigma, Poznań, Poland) in 50 mL of sodium phosphate buffer (DTNB) was added, and the absorbance was checked at 412 nm. A 300 μL volume of the same buffer was used in the control sample. Samples were incubated at 37 °C for 1 h, and the absorbance was checked again at 412 nm. The concentration of sulfhydryl groups was indicated by the difference in absorbance before and after incubation (after subtracting the absorbance of the control). The content was determined using a standard curve prepared with glutathione dilutions (GSH, Sigma, Poznań, Poland), ranging from 0 to 1 mmol/L in 10 mmol/L sodium phosphate buffer (pH 8.0), and expressed in mmol/g protein in homogenate tissue. Each determination was performed in duplicate.

### 2.10. Content of Bityrosine Bridges

The content of bityrosine bridges was measured using a spectrofluorometer according to Rice-Evans et al. [41]. Experimental and control samples were diluted in 0.9% NaCl. All samples were excited with light at 325 nm, and the emission was checked at 410 nm. The spectrofluorometer (Jasco, Tokyo, Japan) was standardized to 100 deflections with quinine sulfate (0.1 µg/mL in 0.1 mol/H_2_SO_4_) at a 350 nm excitation wavelength and a 445 nm emission wavelength. The results were expressed as µg/mg protein in homogenate tissue. Each determination was performed in duplicate.

### 2.11. Content of Formylkynurenine

Formylkynurenine content was measured using a spectrofluorometer as described by Rice-Evans et al. [41]. Experimental and control samples were diluted in 0.9% NaCl. Next, samples were excited with light at 360 nm, and the emission was measured at 454 nm. The spectrofluorometer was standardized as detailed above. The results were expressed as µg/mg protein in homogenate tissue. Each determination was performed in duplicate.

### 2.12. Content of Hydroperoxides

Hydroperoxide content was determined according to Alberti et al. [42]. Each sample in the amount of 20 µL was mixed with 1 mL of 100 mmol/L acetate buffer (pH 4.8). A 10 µL volume of a 3.7 × 10^−1^ M solution of N,N,diethylparaphenylenediamine (DEPPD) was added. After incubation at 37 °C for 90 min, the absorbance was checked at 505 nm against acetate buffer alone. Distilled water was used as a control. The results were recalculated per protein content in the sample and expressed as µmol/g protein in homogenate tissue. Each determination was performed in duplicate.

### 2.13. Statistical Analysis

Statistical analysis of the results was performed using Statistica 13.2 PL software (StatSoft, Krakow, Poland). The data gradient was tested using the Shapiro–Wilk test due to deviation from the normal distribution. The significance of statistical differences (*p* < 0.05) was determined using nonparametric tests. Comparisons between groups I–IV were made using Kruskal–Wallis ANOVA as a nonparametric counterpart of one-way analysis of variance and post hoc tests.

**Figure 1:**
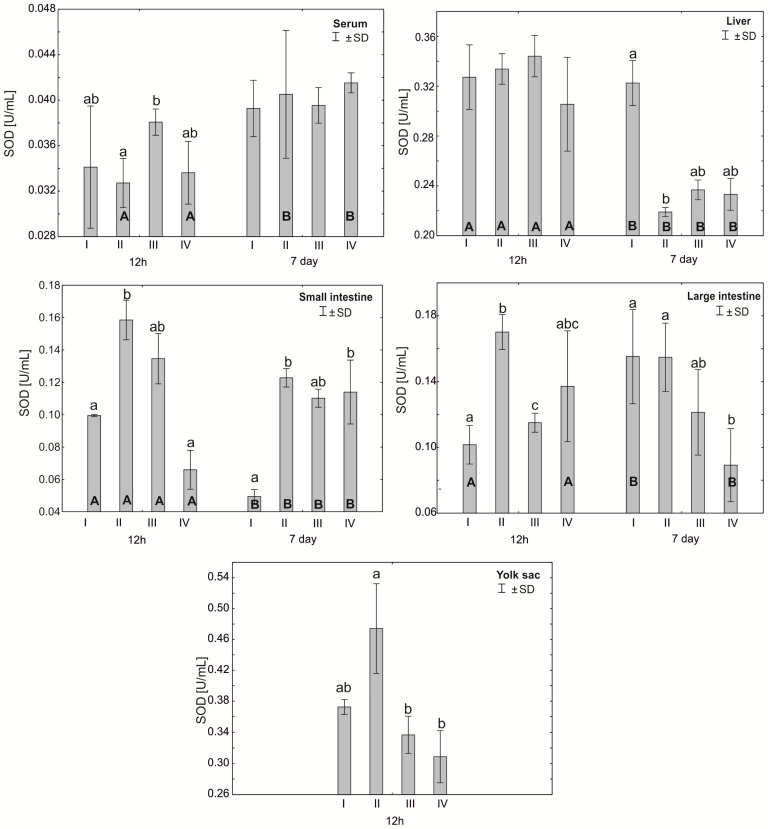
One—way ANOVA and Mann–Whitney U test for superoxide dismutase (SOD) activity in the serum and tissues of broiler chickens at 12 h after hatching and at 7 days of age. The same letter designations indicate no statistically significant differences. Capital letters show statistically significant results (*p* ≤ 0.05) between groups (U test), and lowercase letters indicate differences shown in Kruskal–Wallis ANOVA and post hoc tests. I—control group—eggs injected with sterile 0.9% physiological saline solution; group II—eggs injected with a multi-strain probiotic (1 × 10^5^ CFU *S. cerevisiae*, 1 × 10^7^ CFU *L. casei* and 1 × 10^7^ CFU *L. plantarum*); group III—eggs injected with a multi-strain probiotic (1 × 10^5^ CFU *S. cerevisiae*, 1 × 10^7^ CFU *L. casei* and 1 × 10^7^ CFU *L. plantarum*) and zinc glycine chelate (Zn-Gly); group IV—eggs injected with zinc glycine chelate (Zn-Gly). +/− SD—standard deviation. N = 30.

**Figure 2:**
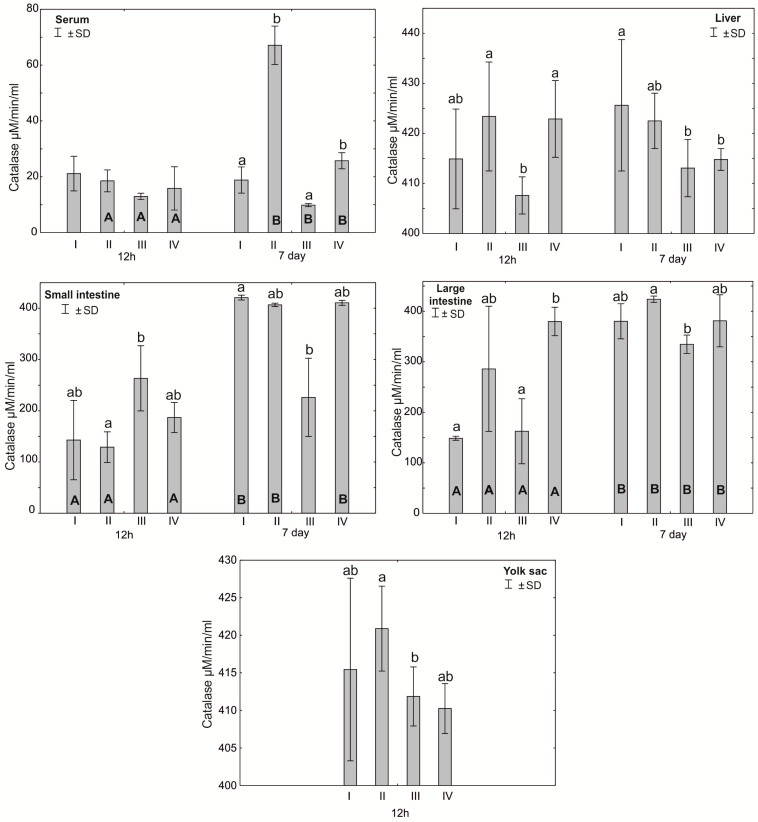
One—way ANOVA and Mann–Whitney U test for catalase (CAT) activity in the serum and tissues of broiler chickens at 12 h after hatching and at 7 days of age. The same letter designations indicate no statistically significant differences. Capital letters show statistically significant results (*p* ≤ 0.05) between groups (U test), and lowercase letters indicate differences shown in Kruskal–Wallis ANOVA and post hoc tests. I—control group—eggs injected with sterile 0.9% physiological saline solution; group II—eggs injected with a multi-strain probiotic (1 × 10^5^ CFU *S. cerevisiae*, 1 × 10^7^ CFU *L. casei* and 1 × 10^7^ CFU *L. plantarum*); group III—eggs injected with a multi-strain probiotic (1 × 10^5^ CFU *S. cerevisiae*, 1 × 10^7^ CFU *L. casei* and 1 × 10^7^ CFU *L. plantarum*) and zinc glycine chelate (Zn-Gly); group IV—eggs injected with zinc glycine chelate (Zn-Gly). +/− SD—standard deviation. N = 30.

**Figure 3:**
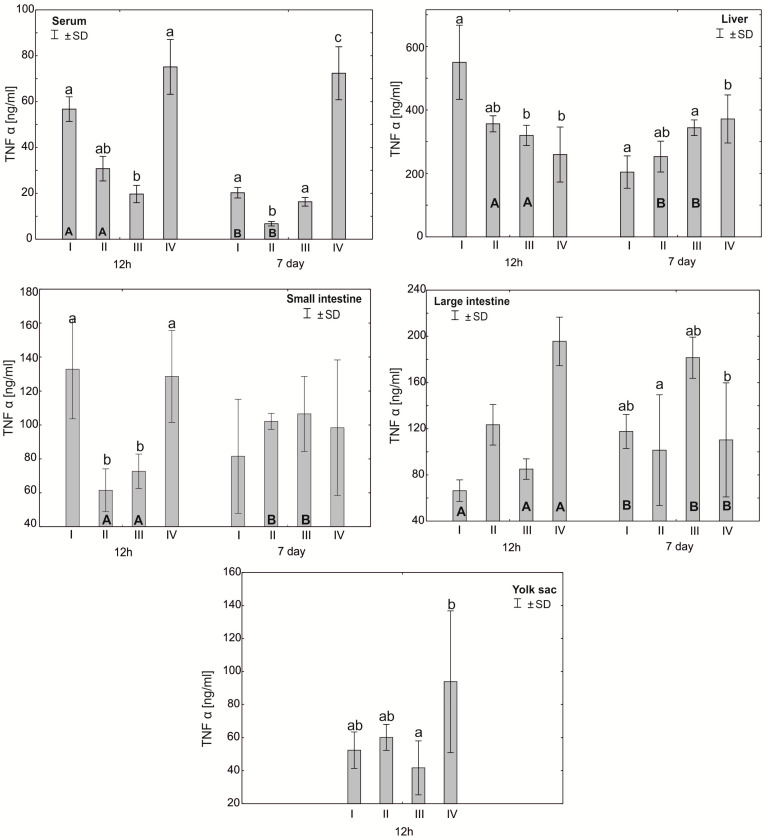
One—way ANOVA and Mann–Whitney U test for tumor necrosis factor α (TNF-α) concentration in the serum and tissues of broiler chickens at 12 h after hatching and at 7 days of age. The same letter designations indicate no statistically significant differences. Capital letters show statistically significant results (*p* ≤ 0.05) between groups (U test), and lowercase letters indicate differences shown in Kruskal–Wallis ANOVA and post hoc tests. I—control group—eggs injected with sterile 0.9% physiological saline solution; group II—eggs injected with a multi-strain probiotic (1 × 10^5^ CFU *S. cerevisiae*, 1 × 10^7^ CFU *L. casei* and 1 × 10^7^ CFU *L. plantarum*); group III—eggs injected with a multi-strain probiotic (1 × 10^5^ CFU *S. cerevisiae*, 1 × 10^7^ CFU *L. casei* and 1 × 10^7^ CFU *L. plantarum*) and zinc glycine chelate (Zn-Gly); group IV—eggs injected with zinc glycine chelate (Zn-Gly). +/− SD—standard deviation. N = 30.

**Figure 4:**
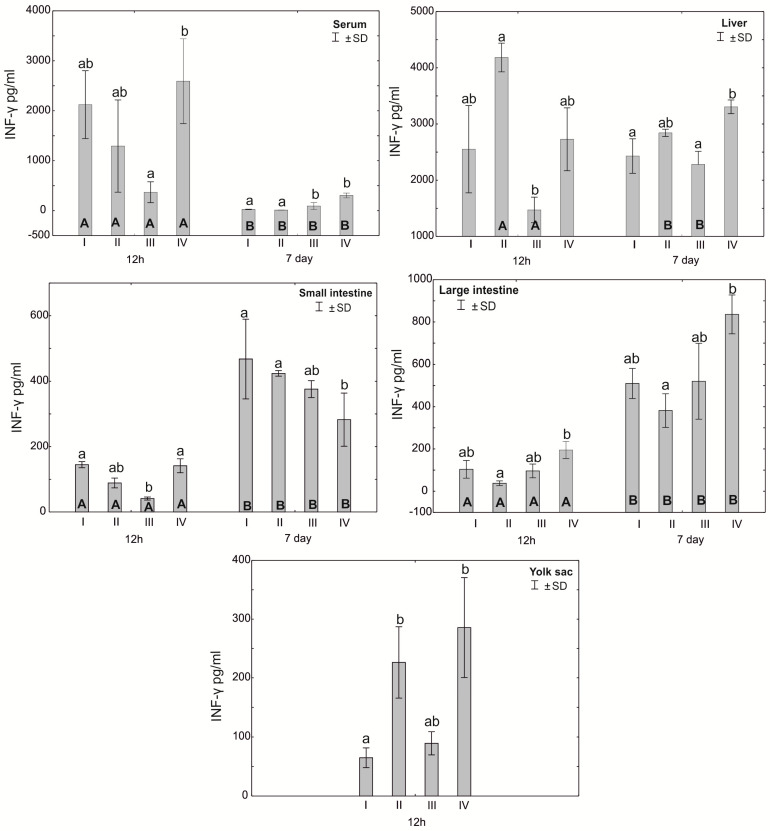
One—way ANOVA and Mann–Whitney U test for interferon γ (IFN−γ) concentration in the serum and tissues of broiler chickens at 12 h after hatching and at 7 days of age. The same letter designations indicate no statistically significant differences. Capital letters show statistically significant results (*p* ≤ 0.05) between groups (U test), and lowercase letters indicate differences shown in Kruskal–Wallis ANOVA and post hoc tests. I—control group—eggs injected with sterile 0.9% physiological saline solution; group II—eggs injected with a multi-strain probiotic (1 × 10^5^ CFU *S. cerevisiae*, 1 × 10^7^ CFU *L. casei* and 1 × 10^7^ CFU *L. plantarum*); group III—eggs injected with a multi-strain probiotic (1 × 10^5^ CFU *S. cerevisiae*, 1 × 10^7^ CFU *L. casei* and 1 × 10^7^ CFU *L. plantarum*) and zinc glycine chelate (Zn-Gly); group IV—eggs injected with zinc glycine chelate (Zn-Gly). +/− SD—standard deviation. N = 30.

**Figure 5:**
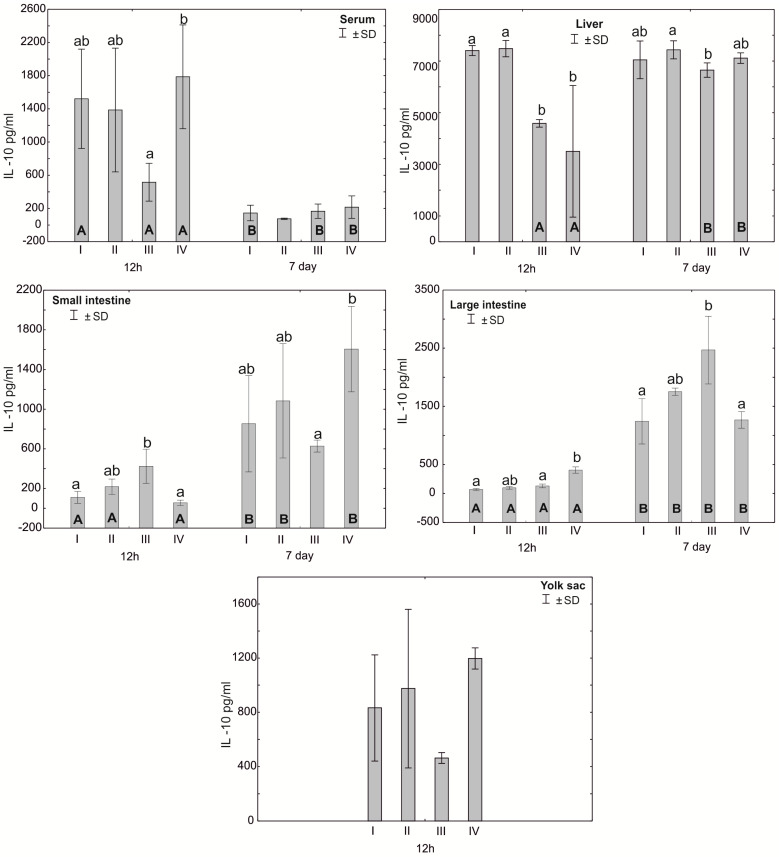
One—way ANOVA and Mann–Whitney U test for interleukin 10 (IL-10) concentration in the serum and tissues of broiler chickens at 12 h after hatching and at 7 days of age. The same letter designations indicate no statistically significant differences. Capital letters show statistically significant results (*p* ≤ 0.05) between groups (U test), and lowercase letters indicate differences shown in Kruskal–Wallis ANOVA and post hoc tests. I—control group—eggs injected with sterile 0.9% physiological saline solution; group II—eggs injected with a multi-strain probiotic (1 × 10^5^ CFU *S. cerevisiae*, 1 × 10^7^ CFU *L. casei* and 1 × 10^7^ CFU *L. plantarum*); group III—eggs injected with a multi-strain probiotic (1 × 10^5^ CFU *S. cerevisiae*, 1 × 10^7^ CFU *L. casei* and 1 × 10^7^ CFU *L. plantarum*) and zinc glycine chelate (Zn-Gly); group IV—eggs injected with zinc glycine chelate (Zn-Gly). +/− SD—standard deviation. N = 30.

**Figure 6:**
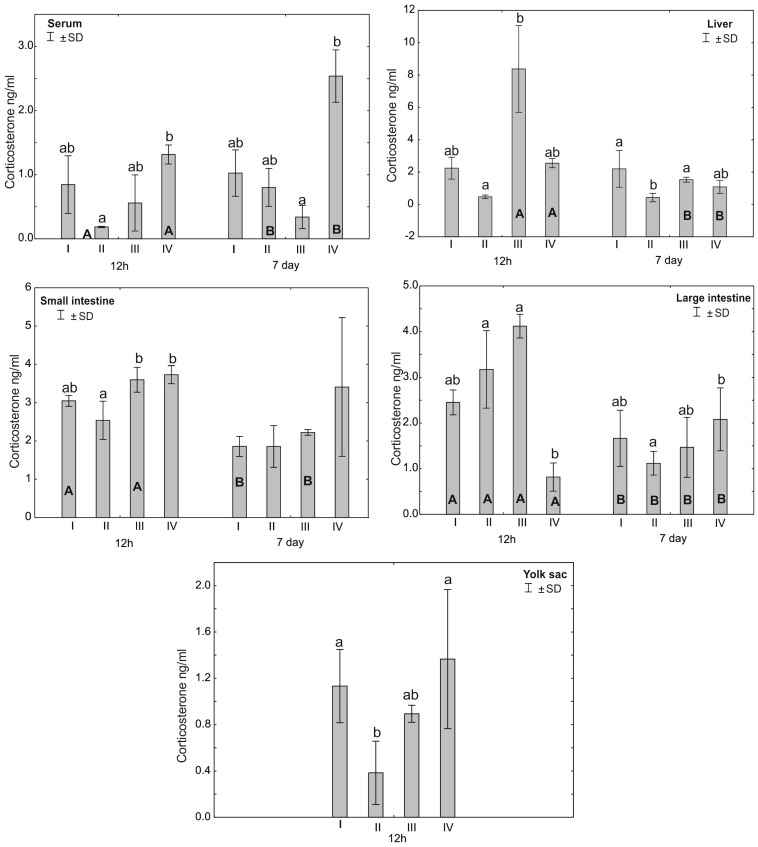
One—way ANOVA and Mann–Whitney U test for corticosterone (CORT) concentration in the serum and tissues of broiler chickens at 12 h after hatching and at 7 days of age. The same letter designations indicate no statistically significant differences. Capital letters show statistically significant results (*p* ≤ 0.05) between groups (U test), and lowercase letters indicate differences shown in Kruskal–Wallis ANOVA and post hoc tests. I—control group—eggs injected with sterile 0.9% physiological saline solution; group II—eggs injected with a multi-strain probiotic (1 × 10^5^ CFU *S. cerevisiae*, 1 × 10^7^ CFU *L. casei* and 1 × 10^7^ CFU *L. plantarum*); group III—eggs injected with a multi-strain probiotic (1 × 10^5^ CFU *S. cerevisiae*, 1 × 10^7^ CFU *L. casei* and 1 × 10^7^ CFU *L. plantarum*) and zinc glycine chelate (Zn-Gly); group IV—eggs injected with zinc glycine chelate (Zn-Gly). +/− SD—standard deviation. N = 30.

**Figure 7:**
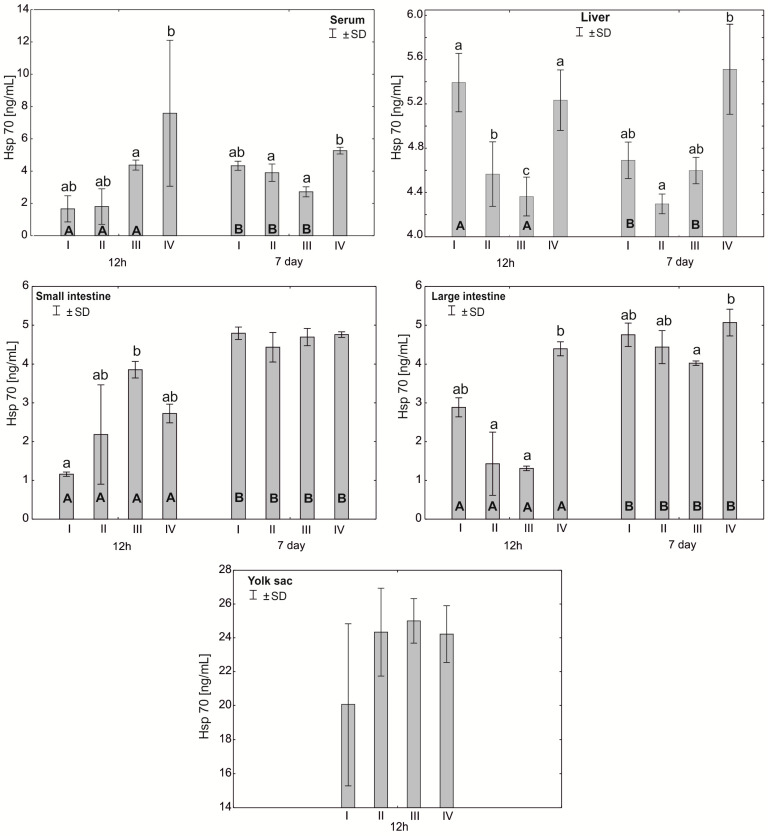
One—way ANOVA and Mann–Whitney U test for heat shock protein (Hsp70) concentration in the serum and tissues of broiler chickens at 12 h after hatching and at 7 days of age. The same letter designations indicate no statistically significant differences. Capital letters show statistically significant results (*p* ≤ 0.05) between groups (U test), and lowercase letters indicate differences shown in Kruskal–Wallis ANOVA and post hoc tests. I—control group—eggs injected with sterile 0.9% physiological saline solution; group II—eggs injected with a multi-strain probiotic (1 × 10^5^ CFU *S. cerevisiae*, 1 × 10^7^ CFU *L. casei* and 1 × 10^7^ CFU *L. plantarum*); group III—eggs injected with a multi-strain probiotic (1 × 10^5^ CFU *S. cerevisiae*, 1 × 10^7^ CFU *L. casei* and 1 × 10^7^ CFU *L. plantarum*) and zinc glycine chelate (Zn-Gly); group IV—eggs injected with zinc glycine chelate (Zn-Gly). +/− SD—standard deviation. N = 30.

The Mann–Whitney U test was used to test the differences within the same group over time, i.e., at 12 h after hatching and at 7 days. The results are presented in graphical form and tables. The same letter designations indicate a lack of statistically significant differences. Capital letters are used to show statistically significant results between groups (U test), and lowercase letters are used to indicate differences shown in Kruskal–Wallis ANOVA and post hoc tests. See Figure 1, Figure 2, Figure 3, Figure 4, Figure 5, Figure 6 and Figure 7 and Table 2 and Table S1. The following designations were used for the variables (experimental samples): I—control group; II—+multi-strain probiotic; III—+multi-strain probiotic + zinc glycine chelate (Zn-Gly); IV—+zinc glycine chelate (Zn-Gly).

## 3. Results

### 3.1. Mean Activity of Superoxide Dismutase (SOD) and Catalase (CAT) in the Serum and Tissues of Chicks

Statistically significant differences were observed in the average superoxide dismutase (SOD) activity in the chicken serum 12 h after hatching between group III and group II. Statistically significant differences in SOD activity between 12 h and 7 days of the study were observed only in groups II and IV. After 7 days of the experiment, statistically significant differences were also observed between groups I and II in the liver cells. After 12 h of the experiment, statistically significant differences were observed in SOD activity in the small intestinal cells in group II compared to groups I and IV. After 7 days, however, statistically significant differences were found in the small intestine between groups III and IV. Statistically significant differences in SOD activity in colon cells after 12 h of the experiment were observed between the control group (I) and groups II and III and between groups II and III. After 7 days, statistically significant differences in SOD activity in the large intestine were observed in group IV compared to groups I and II. The average SOD activity in the yolk sac was statistically significantly different in group II compared to groups III and IV. See Figure 1 and Table S1.

After 7 days, statistically significant differences in serum catalase activity were observed in groups II and IV compared to groups I and III. Statistically significant differences in catalase activity were found in the small intestine after 12 h of the experiment between groups III and II and after 7 days between groups I and III. Catalase activity in the large intestine after 12 h of the experiment was statistically significantly different in groups I and III compared to group IV. After 7 days post-hatch, catalase activity in the large intestine differed statistically significantly between groups III and II. Statistically significant differences in mean catalase activity in the yolk sac were observed between groups II and III. Statistically significant differences in catalase activity in the large intestine were observed in all groups after 7 days of the experiment compared to 12 h after hatching. Statistically significant differences in the activity of this enzyme in the serum, depending on the day of analysis, were observed in groups II–IV. Statistically significant differences in small intestinal catalase activity were observed 7 days after hatching compared to 12 h after hatching in groups I, II and IV (Figure 2, Table S1).

### 3.2. Concentration of Cytokines TNF-α, IFN-γ and IL-10 in the Serum, Liver, Intestines and Yolk Sacs of Chickens

The mean TNF-α concentration in the serum after 12 h of the experiment was statistically significantly different in the control group I and group IV compared to group III. After 7 days of the experiment, the serum concentration of TNF-α showed statistically significant differences in groups I, III and IV compared to group II. The analysis of the TNF-α concentration in the liver after 12 h of the experiment showed statistically significant differences in group I compared to groups III and IV. After 7 days of the experiment, statistically significant differences were found in the concentration of TNF-α in the liver between group IV and groups I and III. In the small intestine, the concentration of TNF-α after 12 h of the experiment differed statistically significantly in groups I and IV compared to groups II and III. The analysis of the concentration of this cytokine in the large intestine after 7 days of the experiment showed statistically significant differences between group II and group III. The concentration of TNF-α in the yolk sac in group III differed statistically significantly from the value obtained in group IV. Depending on the time of sampling, statistically significant differences were observed in the serum TNF-α concentration in groups I and II. The level of this cytokine in the liver in groups II and III also differed statistically significantly after 7 days of the study compared to the values obtained after 12 h. Similarly, the concentration of TNF-α in the small intestine differed statistically significantly in groups II and III compared to the values obtained after 7 days of the experiment. Statistically significant differences in the TNF-α concentration were also observed in the large intestine in groups I, III and IV (Figure 3, Table S1).

The average concentration of IFN-γ in the serum after 12 h of the experiment differed statistically significantly between group IV and group III, and after 7 days, statistically significant differences were found in the serum of birds from groups III and IV compared to the control group (I) and group II. Statistically significant differences in the concentration of IFN-γ in the liver of birds after 12 h of the experiment were observed between groups II and III, while after 7 days, statistically significant differences in this parameter were observed between group IV and groups I and III. The concentration of IFN-γ in the small intestine after 12 h was statistically significantly different in groups I and IV compared to group III. After 7 days of the experiment, the concentration of IFN-γ in the small intestine was statistically significantly different in groups I and II compared to group IV. Statistically significant differences in the concentration of IFN-γ in the large intestine were observed both after 12 h and after 7 days of the experiment between groups II and IV. The concentration of IFN-γ in the yolk sac differed statistically significantly between groups II and IV and group I (control). Statistically significant differences were observed in the concentration of IFN-γ in the serum and in the small and large intestines depending on the measurement time (12 h and 7 days) in all experimental groups. Statistically significant differences in the concentration of IFN-γ were observed in the liver depending on the time of analysis in groups II and III. See Figure 4 and Table S1.

Statistically significant differences in serum IL-10 concentrations were observed after 12 h between group II and group IV. Statistically significant differences were observed in the concentration of IL-10 in the liver after 12 h of the experiment for groups I and II compared to groups III and IV. After 7 days of the experiment, the concentration of IL-10 in the liver was statistically significantly different between groups II and III. The concentration of IL-10 in the small intestine after 12 h of the experiment was statistically significantly different in group III compared to groups I and II. Seven days after hatching, the concentration of IL-10 in the small intestine differed statistically significantly between group IV and group III. The concentration of IL-10 in the large intestine after 12 h of the experiment was statistically significantly different in groups I and II compared to group IV. The concentration of IL-10 in the large intestine 12 h after hatching was statistically significantly different in all groups compared to 7 days after hatching. Statistically significant differences in the concentration of IL-10 in the blood serum were also observed in groups I, III and IV at 7 days post-hatch compared to the concentration obtained 12 h after hatching. The concentration of IL-10 in the liver in groups III and IV after 12 h of the experiment differed statistically significantly from the concentration recorded in these groups 7 days after hatching. In the small intestine, statistically significant differences in the IL-10 concentration were observed depending on the analysis time in groups I, II and IV (Figure 5, Table S1).

### 3.3. Corticosterone (CORT) Concentration in the Serum and Tissues of Chicks

Statistically significant differences in the concentration of corticosterone in the blood serum were observed between group IV and group II after 12 h of the experiment and between group IV and group III after 7 days. In the liver, after 12 h of the experiment, statistically significant differences in the corticosterone concentration were observed between groups II and III. Statistically significant differences in the hormone concentration in the small intestine were noted after 12 h in groups III and IV compared to group II. Statistically significant differences in the corticosterone concentration were observed in the large intestine after 12 h of the experiment in group IV compared to groups III and II. After 7 days of the experiment, statistically significant differences were found in the average corticosterone concentration in the large intestine between groups II and IV. Statistically significant differences were found in the concentration of corticosterone in the yolk sac after 12 h of the experiment in group II compared to groups IV and I. Statistically significant differences in the concentration of corticosterone in the large intestine were observed in all groups in the period from 12 h to 7 days after hatching. Statistically significant differences were observed in the serum corticosterone concentration in groups II and IV after 12 h of the experiment compared to the serum corticosterone level 7 days after hatching. Statistically significant differences in the corticosterone concentration were observed in the liver depending on the day of analysis in groups III and IV (Figure 6, Table S1).

### 3.4. Heat Shock Protein (Hsp70) Concentration in the Serum and Tissues of Chicks

In the serum, the Hsp70 concentration at 12 h post-hatch was statistically significantly different between group IV and group III. After 7 days of the experiment, statistically significant differences were observed in group IV compared to groups II and III. In the liver, statistically significant differences in the Hsp70 concentration after 12 h of the experiment were observed in groups II and III compared to the control group I and in group III compared to groups II and IV. Statistically significant differences were also observed in the large intestine between group IV and groups II and III after 12 h of the experiment. After 7 days of the experiment, statistically significant differences in the Hsp70 concentration in the large intestine were observed between groups IV and III. Statistically significant differences in the Hsp70 concentration between 12 h and 7 days of the experiment were observed in the serum in groups I, II and III and in the liver in groups I and III. In all groups, statistically significant differences in the concentration of the protein were also noted in the small and large intestines between 12 h and 7 days of the study. See Figure 7 and Table S1.

### 3.5. Levels of Total Antioxidant Capacity (T-AOC), Protein, Sulfhydryl (SH) Groups, Bityrosine Bridges, Formylkynurenine and Hydroperoxides/DEPPD in the Serum and Tissues of Chicks

Statistically significant differences were found in the T-AOC of the serum after 12 h between groups III and IV. After 7 days of the experiment, statistically significant differences were observed in the serum T-AOC between groups I and II. Statistically significant differences in the liver after 7 days of the experiment were found in group IV compared to groups I and III. T-AOC analysis in the pectoral muscle showed statistically significant differences in group IV compared to groups II and III after 12 h of the experiment and between groups II and IV after 7 days of the experiment. The analysis of the mean T-AOC in the heart showed statistically significant differences between groups III and IV after 12 h and between groups I and III after 7 days. The mean T-AOC in the small intestine after 12 h of the experiment was statistically significantly different in groups III and II compared to groups I and IV. Statistically significant differences were also observed in the mean T-AOC in the yolk sac after 12 h of the experiment in group I compared to groups II and IV. Statistically significant differences in T-AOC depending on the day of the study (12 h and 7 days) were observed in the serum and pectoral muscle in all experimental groups, in the liver and small intestine in groups II-IV and in the heart in groups III and IV. See Table 2.

Statistically significant differences in the serum protein concentration were noted in group III compared to groups I and II after 12 h of the experiment. After 7 days, statistically significant differences were observed in the serum between groups I and IV. In the liver, the protein level after 12 h was statistically significantly different in groups III and IV compared to groups I and II. In the breast muscle, 12 h after hatching, statistically significant differences in the protein concentration were observed in groups I and IV compared to groups II and III. After 7 days, a statistically significant difference was also found in the average protein concentration in the pectoral muscle between group III and groups I and II. In the heart, the average protein level after 12 h of the experiment was statistically significantly different in groups I and III compared to group II. After 7 days, the protein level in the heart in group III was statistically significantly different from that in groups I and II. In the small intestine, the average protein level after 12 h of the experiment was statistically significantly different in group II compared to groups III and IV. After 7 days of the experiment, the protein level in the small intestine was statistically significantly different in groups II and III compared to group IV. The protein level in the yolk sac differed statistically significantly in group IV compared to group I. Statistically significant differences in protein concentrations between 12 h and 7 days of the study were found in the breast muscle and small intestine in all experimental groups. Statistically significant differences were observed in the serum and liver in groups I, III and IV and in the heart in groups II and IV (Table 2).

Statistically significant differences were observed after 12 h of the experiment in the serum, where the level of SH groups differed statistically significantly in groups I and II compared to groups III and IV. After 7 days, statistically significant differences were observed in the mean serum SH levels in groups I and IV compared to groups II and III. In the liver, after 12 h of the experiment, the level of SH groups differed statistically significantly in group I from the levels in groups II and IV. After 7 days, the level of SH groups in the liver was statistically significantly different in group IV compared to groups I and III. The average level of SH groups in the pectoral muscle was statistically significantly different in group IV compared to groups I and II. In the small intestine, after 12 h of the experiment, the average level of SH groups differed statistically significantly in groups III and IV compared to group I. Statistically significant differences were also found in the average level of SH groups in the yolk sac between group III and groups I and II. Statistically significant differences in the average level of SH groups in the heart were observed after 7 days of the experiment in group II compared to group I. Statistically significant differences in the level of SH groups between 12 h and 7 days of the experiment were observed in groups I, III and IV in the serum and heart; in groups II and IV in the liver; in groups I and II in the pectoral muscle; and in groups II, III and IV in the small intestine (Table 2).

Statistically significant differences were observed in the average content of bityrosine bridges in the serum after 12 h between groups IV and III and in the liver after 12 h in groups I and II compared to groups III and IV. The analysis of the concentration of bityrosine bridges in the liver after 7 days showed statistically significant differences in group I compared to groups II and IV. The average content of bityrosine bridges in the pectoral muscle after 7 days of the experiment was statistically significantly different in group III compared to group I. Statistically significant differences in the heart were observed after 12 h of the experiment between groups I and II and after 7 days in groups II and III compared to group IV. Statistically significant differences in the average content of bityrosine bridges in the small intestine after 12 h of the experiment were observed between group IV and group II. The analysis of the average content of bityrosine bridges in the yolk sac showed statistically significant differences between group I and group III. Statistically significant differences in the average content of bityrosine bridges depending on the day of the study (12 h and 7 days) were observed in the serum and pectoral muscle in groups I, II and IV; in the heart in groups I–III; and in the small intestine in groups II–IV (Table 2).

After 12 h of the experiment, statistically significant differences were observed in the average concentration of formylkynurenine in the liver cells in group IV compared to groups I and II. The average concentration of formylkynurenine in the cells of the small intestine was statistically significantly different in group II compared to groups III and IV. The average concentration of formylkynurenine in the yolk sac in group III differed statistically significantly from the concentration in group I. After 7 days of the experiment, statistically significant differences were observed in all examined tissues except the pectoral muscle. The concentration of the metabolite in the serum differed statistically significantly between group I and groups III and IV. Its average concentration in the liver cells differed statistically significantly in group III compared to groups II and IV. Statistically significant differences were also observed in the concentration of formylkynurenine in the liver between groups II and IV. However, the mean concentration in the heart differed statistically significantly in groups III and IV compared to group I and between groups III and IV. Similarly, the mean concentration of formylkynurenine in the small intestine was statistically significantly different in group III compared to groups I and IV. The analysis of the formylkynurenine concentration within groups depending on the analysis time showed statistically significant differences in the serum in groups I and II and in the pectoral muscle in groups I, II and IV. Similarly, statistically significant differences in the concentration of formylkynurenine in the liver and heart cells were observed in groups II and III and groups I and IV, respectively. The mean concentration of formylkynurenine differed statistically significantly in the small intestine in groups II and IV. See Table 2. Statistically significant differences were observed in the mean DEPPD concentration in the serum after 12 h between group IV and groups I and II. The analysis of the concentration of this compound in the liver after 7 days of the experiment showed statistically significant differences in groups I and II compared to groups III and IV. Differences in the average DEPPD concentration were also observed in the pectoral muscle after 12 h of the experiment in groups III and IV compared to groups I and II. Statistically significant differences in the DEPPD concentration in the heart were observed after 12 h between groups I and IV and after 7 days between groups II and IV. Statistically significant differences were observed in the small intestine after 7 days of the experiment in groups IV and I compared to group III. Statistically significant differences in the DEPPD concentration in the yolk sac after 12 h of the experiment were observed in group I compared to groups III and IV. Statistically significant differences in the DEPPD concentration between the days of analysis (12 h and 7 days) were observed in the liver and small intestine in all experimental groups and in the serum in groups II and IV. Statistically significant differences were also observed between the days of the study in the pectoral muscle in groups I and II and in the heart in groups I–III (Table 2).

## 4. Discussion

During the 21 days of development of the chicken embryo, intensive metabolic changes take place, determining normal growth and development during the embryonic period [43]. Particular importance is ascribed to changes in lipid metabolism during the final period of embryonic development, which lead to the development of oxidative stress [44,45,46]. The multi-strain probiotic used in our experiment contains a mixture of useful beneficial microorganisms, including *Lactobacillus plantarum* and *Lactobacillus casei*. Wang et al. [47] and Hou et al. [48], in a study using pig and mouse models, showed that bacteria of the genus *Lactobacillus* increase SOD activity in the intestines, thereby reducing the ROS concentration and oxidative stress. *Lactobacillus* cells have also been shown to be able to survive in the presence of the hydrogen peroxide they produce, inhibiting the growth of pathogenic microbes [49]. Owing to this property, in ovo administration of *Lactobacillus* bacteria curbs oxidative stress in chicks after they hatch and during their further development.

Our study showed high SOD activity in the small and large intestines of chicks at 12 h post-hatch in the groups supplemented with a multi-strain probiotic and in the small intestine and yolk sac of birds receiving the multi-strain probiotic and Zn-Gly. In the case of CAT, high activity of this enzyme was noted at 12 h post-hatch in the small intestine of birds receiving the multi-strain probiotic and Zn-Gly (group III) and Zn-Gly (group IV) in ovo. An analogous result was obtained for the large intestine of birds receiving the multi-strain probiotic (group II) or Zn-Gly (group IV) in ovo. These results indicate oxidative stress induced by irritation of the intestinal epithelium by the microbes contained in the multi-strain probiotic and by absorption of highly bioavailable zinc chelate. Therefore, the higher SOD activity shown in the intestines post-hatch can be regarded as the effect of these bacteria. It should be noted that at 7 days of age, the SOD activity in the intestines of chicks receiving the multi-strain probiotic and Zn-Gly in ovo was higher than that in the control group. Given the lack of change in SOD activity in the other organs and in the serum, this phenomenon should be linked to local SOD production in the intestines as a result of the irritant effect of zinc glycine chelate on the enterocytes or changes in the intestinal microbiome induced by probiotic and environmental microbes. This is also confirmed by the absence of changes in catalase activity in the intestines.

Analysis of SOD activity in the serum showed an increase at 12 h post-hatch in the group of birds supplemented with a multi-strain probiotic and Zn-Gly. Our results pertaining to this antioxidant enzyme are in contrast to those reported by Shokraneh et al. [46], who showed that in ovo feeding with 500 μg of nano-ZnO on day 17 of embryonic development increases the SOD activity in the serum and its antioxidant activity and reduces oxidative stress in broiler chicks after hatching. These discrepancies may be associated with different means of in ovo administration of Zn, differences in the Zn concentrations in the incubated eggs, and the use of different zinc compounds with different chemical and physical properties than sulfates and oxides. Interesting results were obtained at 7 days of age, when the SOD activity in the liver was significantly higher only in the control group, which suggests activation of this enzyme in response to oxidative stress.

The literature data indicate that zinc plays an important role in the development of the type Th1 immune response, essential to controlling infections through the secretion of a cascade of pro-inflammatory cytokines stimulating immunocompetent effector cells [32,33,50]. This is confirmed by the results of the experiment, in which, following in ovo administration of Zn-Gly chelate, we showed a high concentration of TNF-α in the yolk sac, serum and small and large intestines and of IFN-γ in the yolk sac and serum at 12 h post-hatch. At 7 days of age, a higher TNF-α concentration compared to the control and other experimental groups was noted only in the serum and liver. Stimulation of IFN-γ production in the cells and its release into the serum, shown at 12 h post-hatch, are indicative of the stimulation of T lymphocytes, which increase their cytotoxic activity and promote the cellular phenotype of the Th1 response. Similarly, TNF-α induces the cellular phenotype of the Th1 response. A further indication of immune regulation in response to Zn glycine chelate, limiting the potential occurrence of inflammation, is the high serum concentration of IL-10 at 12 h post-hatch. This cytokine modifies the immune response by acting directly on T cells, inhibiting the synthesis of pro-inflammatory cytokines and limiting the type Th1 immune response.

Different results were obtained following in ovo application of the multi-strain probiotic. In this group, a high concentration of IFN-γ was shown only at 12 h post-hatch in the yolk sac and liver. Similarly, the results for TNF-α did not differ statistically between groups or were significantly lower in the serum, small intestine and liver in comparison to the control group. Similar observations were reported by Pender et al. [51], who, following in ovo feeding with a mixture of probiotics (*Lactobacillus acidophilus*, *Lactobacillus casei*, *Enterococcus faecium* and *Bifidobacterium bifidum*), showed reduced expression of INF-γ, IL-4 and IL-13 in the ileum and caecal tonsils, which are involved in regulation of the innate and acquired immune response. Alizadeh et al. [36] demonstrated that in ovo feeding with 1 × 10^7^ CFU lactobacilli at 18 days of incubation increases the mRNA expression of IFN-α, IFN-β, IL-8, IL-13 and IL-18 in the spleen and the mRNA expression of IFN-γ, IL-2, IL-6, IL-8, IL-12 and IL-18 in the bursa of Fabricius. Shehata et al. [52] reported that in ovo feeding with *B. subtilis* (4 × 10^5^ and 4 × 10^6^ CFU), raffinose (2 and 3 mg) or their combination increases the mRNA expression of IL-2 and toll-like receptor-4 in the ileum. These results clearly indicate that in ovo supplementation with probiotics stimulates the humoral and cellular immune response through regulation of the expression of cytokine genes. Our previous research [34] showed that in ovo administration of a multi-strain probiotic and Zn-Gly chelate causes an increase in the expression of KUL01 in monocytes and macrophages in the spleen and serum, which indicates the activation of these cells and the stimulation of the body’s defense mechanisms.

The antioxidant potential of cells is evaluated by determining total antioxidant capacity (T-AOC) [53,54,55], a useful tool for assessing the antioxidant role of various compounds used as diet supplements for poultry. Our results showed that the use of a multi-strain probiotic (group II) and a multi-strain probiotic with ZnGly chelate (group III) increases T-AOC in the small intestinal tissues at 12 h post-hatch, while no changes in T-AOC were shown in the liver or serum. In addition, T-AOC in the muscle tissue and heart was lower in these groups than in the others. These results suggest that despite the in ovo administration of these supplements, the balance of oxidation and antioxidant processes was not disturbed, and there was no inflammation associated with oxidative stress.

The significant increase in T-AOC shown in the intestinal tissue in the groups of birds receiving a multi-strain probiotic and Zn-Gly in ovo indicates that the probiotic exerts a significant antioxidant effect on rapidly dividing enterocytes during growth and development. The high T-AOC in the yolk sac in all experimental groups indicates that the multi-strain probiotic and Zn-Gly chelate create antioxidant defense mechanisms together with antioxidant substances and antioxidant enzymes contained in the egg.

One of the important metabolic consequences of in ovo injection of a multi-strain probiotic and Zn-Gly was a reduction in the corticosterone concentration in the yolk sac and serum at 12 h post-hatch and in the serum and liver at 7 days of age. Owing to the antioxidant activity of both supplements, free radicals can be effectively scavenged following in ovo administration, and the chicken embryo is less vulnerable to oxidative damage. What is surprising is the high corticosterone concentration in the serum, small intestine and yolk sac at 12 h post-hatch in the groups receiving Zn-Gly. Contrasting results were obtained by Shokraneh et al. [46], who found that chickens subjected to heat stress following in ovo administration of nano-zinc oxide had low corticosterone concentrations despite the increased antioxidant activity of the preparation. The low concentration of glucocorticoids obtained by the researchers was associated with a high total protein concentration in the blood, which was confirmed by Kucuk et al. [56]. In our experiment, however, the protein concentration in the serum and tissues was lower than or comparable to the level in the control group.

Various stress factors responsible for oxidative tissue damage induce the release of ROS, Hsp70 and corticosterone [57]. Hsp70 levels have been shown to be dependent on the stress response and to increase in order to protect cells against its effects [58]. Our study showed a high Hsp70 concentration at 12 h post-hatch in the small intestine of birds in the group receiving the multi-strain probiotic and Zn-Gly chelate in ovo (group III). In the large intestine and serum at 12 h post-hatch and at 7 days of age, the Hsp70 concentration was significantly higher in the group of birds that had received Zn-Gly chelate in ovo (group IV). Contrasting results were reported by Li et al. [59], who showed that zinc can impair the heat shock response in broilers by reducing the mRNA expression of HSP70. Zhang et al. [60], on the other hand, showed that the correct concentration of zinc in mother hens or its additional intake translates to the content of zinc in the eggs, thus reducing concentrations of ROS and HSP70 in the liver of chicken embryos during oxidative stress, while zinc itself plays an antioxidant role by inhibiting the activity of nicotinamide adenine dinucleotide phosphate (NADP). The authors also showed that the addition of Zn-Gly, due to better absorption and utilization of zinc, mitigates the effects of oxidative stress more effectively than inorganic forms [60,61]. In the present study, the higher Hsp70 concentrations in groups III and IV, especially in the intestines, indicate that zinc administered in ovo in combination with glycine is accumulated in excess in the intestinal tissue, leading to metabolic changes in the enterocytes and inducing a stress response as a consequence of rapid cell proliferation. The in ovo application of a multi-strain probiotic also causes the microbes supplied in this manner to interact with microorganisms of the intestinal microbiome and colonize the intestine from the moment of hatching, which can also cause temporary oxidative stress reactions in epithelial cells. Mahmoud et al. [57], in broilers given ascorbic acid, showed a relationship between the synthesis of HSP70 and lipid oxidation in cells subjected to stress. Our results pertaining to concentrations of Hsp70, corticosterone and fatty acid peroxidation products (hydroperoxides/DEPPD) in the serum and tissues suggest that in ovo application of Zn-Gly chelate leads to a temporary stress reaction in newly hatched chicks as an effect of highly bioavailable, easily absorbed zinc in organic form. It is worth mentioning the regulatory role of Hsp70 in the body, manifested as the ability to inhibit inflammation, protecting the organism against debilitating chronic stress [62]. This effect can also be seen in our experiment, in which the concentration of Hsp70 at 7 days of age in chicks that had received the multi-strain probiotic and Zn-Gly chelate in ovo is comparable to or lower than that in the control group. The analysis of the concentration of –SH groups included in this study made it possible to determine the intensity of oxidative protein damage in the chicks. The high concentration of –SH groups in the liver, small intestine and pectoral muscle in the experimental groups at 12 h post-hatch indicates that in ovo application of a multi-strain probiotic and Zn-Gly provides strong antioxidant defense, protecting the embryo and newly hatched chick from harmful external and internal factors. In contrast, a low concentration of SH groups at 12 h post-hatch was noted in the yolk sac and serum in the group receiving the multi-strain probiotic and Zn-Gly (group III) and in the serum in the group receiving Zn-Gly (group IV). These results are indicative of oxidative stress, which may have already occurred in the final stage of embryo development before hatching, when the embryo utilizes the last energy resources of the yolk and intensifies catabolic processes [63]. Alternatively, oxidative stress can result from the hatching process itself, which intensifies it and increases the body’s mobilization to survive this stage and adapt to the surrounding environmental conditions. During this period, proteins used for survival during the “hatch window”, when the newly hatched chick does not yet receive feed, may disintegrate [64]. A higher concentration of –SH groups in the yolk sac was noted only in the group receiving Zn-Gly, which confirms the importance of highly bioavailable zinc in the form of chelates for protein synthesis. On the other hand, at 7 days of age, the concentration of –SH groups in the serum, pectoral muscles, small intestine, liver and heart of poultry that had received the multi-strain probiotic and Zn-Gly in ovo was similar to the level in the control group, which indicates the absence of processes damaging proteins and the maintenance of relative homeostasis of metabolic processes.

It has been demonstrated that the concentration of bityrosine can be used as a marker of disturbances in oxidation processes [65]. We noted a high concentration of bityrosine at 12 h post-hatch in the yolk sac, serum, heart and small intestine of birds that had received the multi-strain probiotic in ovo. It should be emphasized that in the liver of birds receiving the multi-strain probiotic and Zn-Gly, the concentration of bityrosine was lower than that in the control group, which is indicative of efficient antioxidant processes in cells and maintenance of homeostasis of the redox system. Similarly, we showed a higher concentration of formylkynurenine, a product of tryptophan oxidation, at 12 h post-hatch in the yolk sac in all experimental groups and in the small intestine in the group of birds receiving the multi-strain probiotic in ovo. The increase in harmful products of tryptophan metabolism intensifies oxidative stress, which can lead to a strong inflammatory reaction [66]. However, these changes were not observed in the intestine at 7 days, when the concentration of formylkynurenine in the experimental groups was lower than or similar to the level in the control group. Analysis of these relationships should take into account the fact that intensive enterocyte division and apoptosis with the remodeling of structural proteins take place in the intestines, which is linked to the development of birds, resulting in protein damage and ROS generation. The local nature of these changes is evidenced by the low concentration of formylkynurenine in the liver at 12 h post-hatch.

To assess the extent of damage to cell membranes, we used an indirect method, involving the monitoring of a persistent radical cation formed in the reaction of alkoxy and peroxy radicals derived from hydroperoxides with a suitable additive such as DEPPD [67]. The analysis of the results indicated that at 12 h post-hatch, lipid peroxidation is increased in the serum and heart of birds receiving Zn-Gly chelate in ovo and in the pectoral muscle of birds receiving the multi-strain probiotic and Zn-Gly (group III) or Zn-Gly (group IV). This may be due to oxidation processes associated with the body’s response to intensive growth. It is interesting that no increase in lipid peroxidation was shown in the intestines in the experimental groups; furthermore, in the liver of birds receiving the multi-strain probiotic and Zn-Gly in ovo (group III and IV), the intensity of this process was reduced at 7 days post-hatch. The results indicate that a multi-strain probiotic and Zn-Gly chelate applied in ovo have no irritant effect and do not induce inflammation in the intestines, protecting cells against damage.

## 5. Conclusions

In ovo administration of a multi-strain probiotic and zinc glycine chelate increases total antioxidant capacity (T-AOC) and the activity of SOD and CAT. At the same time, the release of pro- and anti-inflammatory cytokines, i.e., TNF-α, IFN-γ and IL-10, indicates the stimulating effects of multi-strain probiotics and Zn-Gly chelate on cellular and humoral immune mechanisms and on processes maintaining the Th1/Th2 balance in chicks during the post-hatch period and the first few days of life. In ovo administration of Zn-Gly chelate can induce a local stress response in the intestines, manifested as increased lipid peroxidation and protein oxidation. The results of our research suggest that the combined in ovo administration of a multi-strain probiotic and Zn-Gly chelate protects developing embryos and newly hatched chicks against oxidative stress, but this should be confirmed by studies using a challenger. An additional advantage of the use of a multi-strain probiotic and zinc glycine chelate, whose beneficial effects were demonstrated in our study, is that the production cost of this mixture is relatively low. However, the feasibility of the routine use of the preparation is limited by the labor-intensive and time-consuming in ovo administration. Further research on the effects of multi-strain probiotics and zinc glycine chelates on metabolic processes taking place in cells at the level of the nucleus and cytosol will help to elucidate unknown aspects of antioxidant protection.

## Figures and Tables

**Table 1:** In ovo supplementation with a multi-strain probiotic and Zn-Gly chelate.

Group	Replicates/Number of Eggs per Replicate N=	Volume and Solution of Bioactive Compounds Injected In Ovo at 17 DOI *
I—control group	10/35 350	500 µL 0.9% NaCl
II	10/35 350	100 µL multi-strain probiotic + 400 µL MQ water
III	10/35 350	100 µL multi-strain probiotic + 100 µL Zn-Gly + 300 µL MQ water
IV	10/35 350	100 µL Zn-Gly + 400 µL MQ water

* DOI—day of incubation; N—number of eggs in all replicates; MQ—Milli-Q water. I—control group—eggs injected with sterile 0.9% isotonic sodium chloride solution; group II—eggs injected with a multi-strain probiotic (100 µL of multi-strain probiotic contains 1 × 10^5^ CFU *S. cerevisiae*, 1 × 10^7^ CFU *L. casei* and 1 × 10^7^ CFU *L. plantarum*); group III—eggs injected with a multi-strain probiotic (100 µL of multi-strain probiotic contains 1 × 10^5^ CFU *S. cerevisiae*, 1 × 10^7^ CFU *L. casei* and 1 × 10^7^ CFU *L. plantarum)* and zinc glycine chelate (Zn-Gly); group IV—eggs injected with zinc glycine chelate (Zn-Gly).

**Table 2:** Levels of total antioxidant capacity (T-AOC), protein, sulfhydryl (SH) groups, bityrosine bridges, formylkynurenine and hydroperoxides/DEPPD in the serum and tissues of chicks.

Time	Tissue		Formylkynurenine (μg/mg Protein in Homogenate Tissue)				Sulfhydryl Groups/SH (mmol/g Protein in Homogenate Tissue)			
		N=	Groups							
			I	II	III	IV	I	II	III	IV
12 h	Serum	30	**0.21 ± 0.02**	**0.26 ± 0.05**	0.23 ± 0.06	0.21 ± 0.01	10.41 ± 0.43 a ***	11.27 ± 0.49 a ***	8.74 ± 0.37 b ***	9.59 ± 0.46 b ***
	Liver		1.10 ± 0.44 a ***	**0.76 ± 0.02 a** ***	**0.51 ± 0.03 ab** ***	0.44 ± 0.01 b ***	62.82 ± 1.34 a ***	89.14 ± 0.20 b ***	72.0 ± 2.15 ab ***	80.33 ± 2.92 b ***
	Pectoral muscle		**0.42 ± 0.04**	**0.41 ± 0.14**	0.29 ± 0.12	**0.42 ± 0.07**	83.5 ± 6.70 a **	77.74 ± 0.49 a **	94.27 ± 5.15 ab **	98.08 ± 3.46 b **
	Heart		**0.35 ± 0.09**	0.45 ± 0.08	0.34 ± 0.05	**0.38 ± 0.03**	**66.49 ± 12.36**	73.08 ± 5.69	**67.49 ± 2.21**	**73.97 ± 2.81**
	Small intestine		0.63 ± 0.09 ab ***	1.27 ± 0.05 a ***	0.47 ± 0.14 b ***	**0.30 ± 0.01 b** ***	67.98 ± 0.57 a ***	83.65 ± 4.29 ab ***	94.81 ± 5.62 b ***	**86.78 ± 5.06 b** ***
	Yolk sac		1.23 ± 0.35 b **	1.64 ± 0.50 ab **	2.59 ± 0.21 a **	2.0 ± 0.66 ab **	20.33 ± 3.49 a **	22.62 ± 0.51 a **	13.55 ± 2.8 b **	25.10 ± 6.59 ab **
After 7 days	Serum	30	0.13 ± 0.03 a *	0.16 ± 0.04 abc *	0.17 ± 0.02 * c	0.19 ± 0.03 b *	11.8 ± 0.68 a *	10.31 ± 0.93 b *	10.72 ± 0.34 b *	11.91 ± 1.28 a *
	Liver		0.78 ± 0.03 abc ***	0.56 ± 0.09 b ***	0.87 ± 0.08 a ***	0.53 ± 0.20 c ***	67.58 ± 5.06 a **	71.91 ± 12.33 ab **	63.61 ± 10.57 a **	86.61 ± 1.82 b **
	Pectoral muscle		**0.22 ± 0.05**	**0.18 ± 0.01**	0.3 ± 0.18	**0.24 ± 0.08**	**101.27 ± 2.88**	**102.26 ± 3.24**	101.85 ± 5.27	103.29 ± 6.64
	Heart		0.52 ± 0.06 a ***	0.37 ± 0.03 abc ***	0.34 ± 0.00 c ***	0.5 ± 0.04 b ***	95.56 ± 1.07 a ***	75.04 ± 8.77 b ***	88.84 ± 1.88 ab ***	88.76 ± 1.66 ab ***
	Small intestine		0.58 ± 0.11 a ***	0.43 ± 0.05 ab ***	0.34 ± 0.02 b ***	**0.53 ± 0.03 a** ***	70.5 ± 3.73	**71.05 ± 6.56**	**70.75 ± 2.24**	**74.06 ± 0.15**
Time	Tissue		Bityrosine bridges (µg/mg protein in homogenate tissue)				T-AOC (μmol/g protein in homogenate tissue)			
		N=	Groups							
			I	II	III	IV	I	II	III	IV
12 h	Serum	30	0.73 ± 0.10 ab *	0.86 ± 0.13 ab *	0.61 ± 0.15 a *	0.87 ± 0.08 b *	88.37 ± 7.00 ab ***	94.65 ± 2.72 ab ***	111.8 ± 7.60 a ***	69.79 ± 1.38 b ***
	Liver		6.87 ± 2.24 a ***	6.88 ± 0.19 a ***	3.77 ± 0.15 b ***	3.87 ± 0.05 b ***	56.07 ± 22.62	**42.65 ± 5.20**	**40.76 ± 5.30**	**57.83 ± 5.48**
	Pectoral muscle		**3.79 ± 1.43**	**4.31 ± 1.90**	3.26 ± 2.03	**3.19 ± 0.43**	31.38 ± 1.11 ab ***	23.66 ± 2.46 b ***	23.45 ± 1.61 b ***	32.40 ± 0.61 a ***
	Heart		2.68 ± 0.21 a *	3.72 ± 0.64 b *	3.19 ± 0.75 ab *	3.62 ± 0.57 ab *	26.47 ± 3.16 ab *	25.12 ± 2.44 ab *	24.20 ± 1.12 b *	30.83 ± 2.68 a *
	Small intestine		5.58 ± 1.90 ab ***	9.21 ± 2.99 a ***	4.14 ± 1.07 ab ***	2.75 ± 0.29 b ***	57.31 ± 6.56 a ***	99.75 ± 13.51 b ***	97.53 ± 14.11 b ***	54.48 ± 1.98 a ***
	Yolk sac		4.44 ± 0.39 a **	5.94 ± 1.54 ab **	7.31 ± 0.40 b **	6.79 ± 0.22 ab **	21.38 ± 1.01 a **	45.10 ± 11.07 b **	44.19 ± 7.84 ab **	49.96 ± 6.81 b **
After 7 days	Serum	30	**0.32 ± 0.16**	**0.46 ± 0.02**	0.5 ± 0.11	**0.46 ± 0.06**	30.05 ± 2.18 a *	44.13 ± 0.55 b *	37.14 ± 2.99 ab *	44.73 ± 14.51 ab *
	Liver		5.38 ± 0.15 a ***	3.30 ± 0.70 b ***	4.02 ± 0.43 ab ***	1.92 ± 0.50 b ***	77.95 ± 5.96 a **	82.30 ± 16.71 ab ***	78.61 ± 9.84 a ***	108.71 ± 6.29 b ***
	Pectoral muscle		1.92 ± 0.11 a *	2.12 ± 0.20 ab *	2.48 ± 0.30 b *	2.19 ± 0.33 ab *	18.16 ± 1.85 ab ***	15.71 ± 0.17 b ***	19.04 ± 0.26 ab ***	24.76 ± 2.66 a ***
	Heart		2.30 ± 0.23 ab ***	1.59 ± 0.70 a ***	1.53 ± 0.12 a ***	3.99 ± 0.24 b ***	26.61 ± 0.66 a *	24.97 ± 1.55 ab *	22.30 ± 0.71 b *	24.37 ± 3.16 ab *
	Small intestine		3.85 ± 0.45	**3.00 ± 0.34**	**2.39 ± 0.10**	**3.88 ± 0.24**	55.63 ± 8.46 ab *	61.96 ± 8.85 a *	58.57 ± 1.65 ab *	48.38 ± 1.69 b *
Time	Tissue		Protein (g/L in homogenate tissue)				Hydroperoxides/DEPPD (µmol/g protein in homogenate tissue)			
		N=	Groups							
			I	II	III	IV	I	II	III	IV
12 h	Serum	30	25.71 ± 0.68 a **	25.93 ± 1.03 a **	29.28 ± 1.57 b **	27.9 ± 0.72 ab **	0.04 ± 0.01 a **	0.04 ± 0.01 a **	0.05 ± 0.02 ab **	0.10 ± 0.01 b **
	Liver		51.41 ± 2.00 a ***	47.34 ± 7.90 a ***	65.52 ± 4.98 b ***	63.46 ± 2.33 b ***	**0.28 ± 0.01**	**0.27 ± 0.01**	**0.27 ± 0.03**	**0.29 ± 0.01**
	Pectoral muscle		6.36 ± 0.18 a ***	8.57 ± 1.08 b ***	11.56 ± 0.87 b ***	7.06 ± 0.18 a ***	0.15 ± 0.03 a ***	0.18 ± 0.01 a ***	0.25 ± 0.02 b ***	0.23 ± 0.02 b ***
	Heart		12.49 ± 2.59 a **	8.36 ± 0.55 b **	12.37 ± 0.12 a **	9.76 ± 0.88 ab **	0.18 ± 0.00 a ***	0.20 ± 0.01 ab ***	0.22 ± 0.03 ab ***	0.26 ± 0.01 b ***
	Small intestine		7.51 ± 1.83 ab ***	5.49 ± 1.12 a ***	15.45 ± 1.21 b ***	15.37 ± 0.25 b ***	**0.21 ± 0.001**	**0.20 ± 0.01**	**0.20 ± 0.01**	**0.21 ± 0.01**
	Yolk sac		41.26 ± 0.17 a ***	26.45 ± 9.67 ab ***	19.05 ± 0.35 ab ***	10.95 ± 3.67 b ***	0.20 ± 0.001 a ***	0.19 ± 0.01 ab ***	0.18 ± 0.003 b ***	0.18 ± 0.01 b ***
After 7 days	Serum	30	27.33 ± 1.07 a *	26.51 ± 1.86 ab *	26.94 ± 0.45 ab *	24.76 ± 0.75 b *	0.07 ± 0.03 *	0.09 ± 0.04 *	0.05 ± 0.006 *	0.06 ± 0.02 *
	Liver		**37.09 ± 7.99**	39.25 ± 7.06	**38.77 ± 10.37**	**28.84 ± 2.00**	0.37 ± 0.01 a ***	**0.32 ± 0.002 a** ***	**0.30 ± 0.01 b** ***	0.31 ± 0.01 b ***
	Pectoral muscle		22.08 ± 2.29 a ***	**24.07 ± 1.49 a** ***	**14.38 ± 0.27 b** ***	**20.51 ± 0.37 ab** ***	**0.23 ± 0.02**	**0.23 ± 0.01**	0.22 ± 0.03	0.21 ± 0.005
	Heart		10.38 ± 0.19 a **	10.31 ± 1.53 a **	12.62 ± 0.46 b **	11.90 ± 1.06 ab **	0.30 ± 0.01 ab **	0.32 ± 0.01 a **	0.30 ± 0.002 ab **	0.28 ± 0.01 b **
	Small intestine		23.32 ± 1.64 ab **	29.73 ± 6.81 a **	25.98 ± 1.26 a **	20.94 ± 1.48 b **	0.24 ± 0.00 a ***	0.23 ± 0.00 ab ***	0.22 ± 0.01 b ***	0.25 ± 0.003 a ***

Values are expressed as mean and standard deviation (±SD). One–way ANOVA and the Mann–Whitney U test were used to show the statistical significance of differences in the serum and tissues of broiler chickens at 12 h after hatching and at 7 days of age. Level of statistical significance: * *p* < 0.05, ** *p* < 0.001, *** *p* < 0.0001. Lowercase letters (a, b, c) indicate differences shown in Kruskal–Wallis ANOVA and post hoc tests. The same letter designations indicate no statistically significant differences. Significant differences in the Mann–Whitney U test between groups at the analysis times are shown in bold. I—control group—eggs injected with sterile 0.9% physiological saline solution; group II—eggs injected with a multi-strain probiotic (1 × 10^5^ CFU *S. cerevisiae*, 1 × 10^7^ CFU *L. casei* and 1 × 10^7^ CFU *L. plantarum*); group III—eggs injected with a multi-strain probiotic (1 × 10^5^ CFU *S. cerevisiae*, 1 × 10^7^ CFU *L. casei* and 1 × 10^7^ CFU *L. plantarum*) and zinc glycine chelate (Zn-Gly); group IV—eggs injected with zinc glycine chelate (Zn-Gly). SD—standard deviation. N—number of samples of serum, liver, pectoral muscle, heart, small intestine and yolk sac tested in each group at 12 h after hatching and at 7 days of age.

## Data Availability

All data generated or analyzed during this study are included in this published article and are available on request from the corresponding author.

## Supplementary Materials

The following supporting information can be downloaded at: https://www.mdpi.com/article/10.3390/antiox12111905/s1, Supplementary Table S1: Comparison of the concentrations of IL-10, IFN-γ, TNF-α, corticosterone and Hsp 70 and the mean activity of catalase and superoxide dismutase (SOD) in chicken serum and tissues.
