# Supplementary material for: Effect of In Ovo Administration of a Multi-Strain Probiotic and Zinc Glycine Chelate on Antioxidant Capacity and Selected Immune Parameters in Newly Hatched Chicks
Source: Antioxidants (Basel). 2023 Oct 24;12(11):1905. doi: 10.3390/antiox12111905 (PMC10669093; doi:10.3390/antiox12111905)
Supplement: Supplementary file 1 [file antioxidants-12-01905-s001.zip › antioxidants-2640336-supplementary.pdf]

Supplementary Table S1: Comparison of the concentrations of IL-10, IFN-γ, TNF-α, corticosterone, and Hsp 70, and the mean activity of catalase and superoxide dismutase (SOD) in chicken serum and tissues.

Parameters	Group	N =	12 hours					7 days			
			Serum	Liver	Small intestine	Large intestine	Yolk sac	Serum	Liver	Small intestine	Large intestine
Corticosterone ng/ml	I	30	0.85±0.41***	2.24±0.62***	3.05±0.13**	2.45±0.25***	1.13±0.29**	1.03±0.33***	2.20±1.04**	1.86±0.24	1.67±0.56**
	II		0.19±0.01***	0.47±0.10***	2.54±0.46**	3.17±0.77***	0.38±0.25**	0.8±0.27***	0.43±0.24**	1.86±0.50	1.12±0.24**
	III		0.56±0.40***	8.38±2.45***	3.60±0.30**	4.12±0.24***	0.89±0.07**	0.34±0.16***	1.53±0.14**	2.22±0.07	1.47±0.60**
	IV		1.32±0.14***	2.55±0.26***	3.73±0.21**	0.82±0.28***	1.37±0.55**	2.54±0.37***	1.08±0.37**	3.41±1.65	2.08±0.63**
SOD U/ml	I	30	0.034±0.0049*	0.327±0.0237	0.1±0.0006***	0.102±0.0107**	0.372±0.0087***	0.039±0.0023	0.323±0.0165***	0.049±0.0039***	0.155±0.0261**
	II		0.033±0.002*	0.334±0.0112	0.159±0.0112***	0.17±0.0097**	0.474±0.0531***	0.041±0.0051	0.219±0.0033***	0.123±0.0052***	0.155±0.0189**
	III		0.038±0.0011*	0.344±0.0151	0.135±0.0142***	0.115±0.0053**	0.337±0.0217***	0.040±0.0014	0.237±0.0072***	0.11±0.0051***	0.121±0.0238**
	IV		0.034±0.0025*	0.306±0.0344	0.066±0.011***	0.137±0.0307**	0.309±0.0306***	0.042±0.0008	0.233±0.0117***	0.114±0.018***	0.089±0.0203**
Catalase µM/min/ml	I	30	21.14±5.66	414.91±9.10*	142.9±70.71*	148.73±3.66**	415.45±11.10*	18.83±4.28***	425.63±11.99**	420.86±4.33***	380.12±31.69**
	II		18.53±3.58	423.39±9.93*	129.08±27.32*	286.13±113.01**	420.89±5.16*	67.11±6.28***	422.51±5.05**	406.63±3.06***	423.90±5.65**
	III		12.96±1.04	407.62±3.39*	263.39±57.99*	162.62±58.83**	411.85±3.59*	9.85±0.52***	413.08±5.22**	226.15±69.65***	334.62±16.63
	IV		15.83±7.08	422.9±70*	186.9±26.84*	379.73±25.73**	415.45±11.10*	25.74±2.63***	414.8±1.98**	410.64±4.43***	381.09±47.07
Hsp70 ng/ml	I	30	1.66±0.74**	5.39±0.24***	1.16±0.05**	2.89±0.23***	20.06±4.36	4.33±0.26	4.69±0.15***	4.79±0.15***	4.76±0.28**
	II		1.8±1.01**	4.57±0.27***	2.18±1.17**	1.43±0.74***	24.34±2.38	3.9±0.49	4.3±0.08***	4.43±0.35***	4.44±0.39**
	III		4.38±0.28**	4.36±0.16***	3.86±0.19**	1.31±0.05***	25±1.20	2.72±0.28	4.60±0.11***	4.69±0.2***	4.03±0.05**
	IV		7.58±4.12**	5.23±0.25***	2.73±0.22**	4.39±0.16***	24.22±1.53	5.27±0.19	5.51±0.37***	4.76±0.07***	5.07±0.32**
TNF-α ng/ml	I	30	56.73±4.90**	550.2±106.76***	132.87±26.70***	66.35±8.56	52.41±10.06***	20.27±2.09***	204.23±46.52***	81.56±30.69	117.72±13.49**
	II		30.76±4.89**	356.34±23.12***	61.54±11.50***	123.5±16.06	60.14±7.14***	6.76±0.89***	253.08±44.32***	102.13±4.30	101.48±43.84**
	III		19.69±3.43**	319.96±29.16***	72.67±9.29***	85.08±8.10	41.7±14.90***	16.32±1.73***	343.97±22.48***	106.55±20.18	181.5±16.24***
	IV		75.13±10.89**	259.55±79.18***	128.65±24.72***	195.68±19.1	93.84±39.19***	72.37±10.56***	371.58±69.12***	98.33±36.45	110.35±45.11**

**IL -10 pg/ml**	**I**		**1521.42±546.46***	7407.56±176.91***	**110.7±54.99*****	**67.84±17.08*****	832.22±357.25	**145.34±85.59**	7046.02±667.56*	**853.59±444.08***	**1242.18±356.25****
	**II**	30	1386.39±680.52*	7483.41±289.48***	**217.88±70.55*****	**96.96±21.9*****	975.73±534.26	75.2±7.79	7435.1±320.29*	**1083.95±525.65***	**1752.28±57.94****
	**III**		**516.19±208.23***	**4586.4±134.03*****	424.24±157.14***	**129.41±29.9*****	463.41±36.41	**121.26±97.74**	**6650.28±255.04***	627.9±54.88*	**2468.45±531.25****
	**IV**		**1786.3±571.19***	**3504.83±2327.4*****	**55.45±24.65*****	**402.92±49.79*****	1197.24±71.54	**215.9±123.76**	**7115.58±185.92***	**1605.8±392.85***	**1266.58±129.41****
**INF γ pg/ml**	**I**		**2122.28±620.21****	2551.24±709.07***	**144.67±8.49*****	**103.84±38.32*****	64.83±15.23***	**25.26±6.08*****	2429.4±280.1***	**467.7±111.08****	**509.9±65.11****
	**II**	30	**1293.09±842.93****	**4183.56±232.94*****	**88.73±13.73*****	**37.84±10.17*****	226.25±55.48***	**11.82±1.57*****	**2841.97±58.9*****	**424.03±7.75****	**381.61±72.66****
	**III**		**367.73±191.37****	**1470.88±207.9*****	**41.11±4.2*****	**96.11±29.73*****	89.28±17.89***	**92.14±64.37*****	**2280.03±213.1*****	**376.04±23.69****	**519.96±163.62****
	**IV**		**2591.68±775.47****	2728.03±510.74***	**141.4±19.49*****	**195.15±37.25*****	285.59±77.69***	**304.65±43.61*****	3305.02±111.75***	**282.48±74.27****	**835.91±84****

Values are expressed as mean and standard deviation (±SD). One-way ANOVA and the Mann–Whitney U test were used to show the statistical significance of differences in the serum and tissues of broiler chickens at 12 hours after hatching and at 7 days of age. Level of statistical significance: * — p <0.05, ** — p <0.001, *** — p <0.0001. Lower-case letters (a, b, c) indicate differences shown in Kruskal–Wallis ANOVA and post-hoc tests. The same letter designations indicate no statistically significant differences. Significant differences in the Mann–Whitney U test between groups at the analysis times are shown in bold. I - control group – eggs injected with sterile 0.9% physiological saline solution; group II - eggs injected with a multi-strain probiotic (1×10^5 CFU *S. cerevisiae*, 1×10^7 CFU *L. casei*, and 1×10^7 CFU *L. plantarum*); group III - eggs injected with a multi-strain probiotic (1×10^5 CFU *S. cerevisiae*, 1×10^7 CFU *L. casei*, and 1×10^7 CFU *L. plantarum*) and zinc glycine chelate (Zn-Gly); group IV - eggs injected with zinc glycine chelate (Zn-Gly). SD - standard deviation. N - number of samples of serum, liver, pectoral muscle, heart, small intestine and yolk sac, tested in each group at 12 hours after hatching and at 7 days of age.
